# Normative and self-perceived orthodontic treatment need of a Peruvian university population

**DOI:** 10.1186/1746-160X-2-22

**Published:** 2006-08-03

**Authors:** Eduardo Bernabé, Carlos Flores-Mir

**Affiliations:** 1 Profesor Asociado, Departamento Académico de Odontología Social, Facultad de Estomatología, Universidad Peruana Cayetano Heredia, Lima, Peru; 2 Unidad de Investigación en Salud Pública Dental, Universidad Peruana Cayetano Heredia, Lima, Peru; 3 Clinical Associate Professor, Orthodontic Graduate Program, Department of Dentistry, University of Alberta, Edmonton, Canada

## Abstract

**Background:**

Previous studies on orthodontic treatment need in young adults have shown that up to 50% had malocclusions that needed orthodontic treatment. The aims of this study were to assess the normative and self-perceived need for orthodontic treatment using the Index of Orthodontic Treatment Need (IOTN) and to determine if the treatment need levels were influenced by sex, age and socio-economic status (SES) in a sample of Peruvian young adults.

**Methods:**

281 first-year students (157 male and 124 female students) with a mean age of 18.1 +/- 1.6 years were randomly selected and evaluated through the Dental Health Component (DHC) and Aesthetic Component (AC) of the IOTN. Structured interview and clinical examination were used to assess the students. Descriptive statistics and Chi-square tests were used for data analysis with statistical significance set at *P *< 0.05.

**Results:**

An intra-examiner reliability of 0.89 was obtained (weighted Kappa). The percentage of students according to SES was 51.2%, 40.6% and 8.2% corresponding to low, medium and high SES respectively. The percentage of students with DHC grades 4–5 was 29.9% whereas the percentage of students with AC grades 8–10 was 1.8%. There were no significant differences in the distribution of normative and self-perceived orthodontic treatment need based on sex, age and SES comparisons.

**Conclusion:**

Normative orthodontic treatment need was not matched by a similar level of self-perceived treatment need in these young adults. Sex, age and SES were non-significant factors associated with levels of treatment need.

## Background

The planning of orthodontic treatment within a public health system requires information on the orthodontic treatment needs of the population [1]. This would permit selection of cases to be treated based on financial, political or administrative purposes [2]. These indexes quantify and summarize a set of clinical and/or radiological data to obtain a final quantitative score or qualitative categorization [3-5]. In essence, the primary purpose of an orthodontic treatment need index is to identify individuals who would benefit from orthodontic treatment and would be given treatment priority [5].

The assessment of orthodontic treatment need, based on the Index of Orthodontic Treatment Need (IOTN) [6], has gained international acceptance in recent years because it was found to be valid, reliable and easy to use [3,4,7-11].

Briefly, the IOTN has two parts: the Aesthetic (AC) and the Dental Health (DHC) components. The AC assesses the perception of an individual on the attractiveness of his/her dentition through a 10-point photographical scale showing different levels of dental attractiveness, with photo 1 representing the most attractive and photo 10 the least attractive [6,12]. Photos 1 to 4 represent 'no need for treatment'; 5 to 7 'borderline need for treatment' and 8 to 10 'definite need for orthodontic treatment'. The DHC assesses 10 traits of malocclusion: overjet, reverse overjet, overbite, openbite, crossbite, crowding, impeded eruption, defects of cleft lip and palate as well as any craniofacial anomaly, Class II and Class II buccal occlusions, and hypodontia. The DHC identifies the worst occlusal trait that is potentially detrimental to dental health and each given grade is a reflection of the level of orthodontic treatment need for the basis of treatment prioritization [3,6,7,13]. Grades 1 and 2 represent 'no need for treatment'; grade 3 'moderate/borderline need for treatment' and grades 4 and 5 'definite need for orthodontic treatment.

Since consciousness of body image increases during childhood and adolescence, young adults are considered to be a relevant age group for the study of personal dental appearance perception [14]. The determination of prevailing orthodontic treatment need in young adults is important because individuals with high treatment needs can be identified and advised accordingly [15]. In addition, information regarding orthodontic treatment need of young adults would be relevant for evaluating satisfaction with dental appearance in orthodontically treated and untreated individuals and for assessing treatment outcomes [14,16].

Although studies on young adults have reported that up to 50% of study samples had definite orthodontic treatment needs [15,17-28], there is little information regarding treatment need levels in developing countries such as Peru. Nevertheless, malocclusion is undoubtedly a public health concern in any country.

The aims of this study were to assess the normative and self-perceived need for orthodontic treatment in a sample of Peruvian young adults using the IOTN and to compare the treatment need levels according to sex, age and socio-economic status (SES) of the students. This should serve as a starting point for an adaptation or the creation of a new index for orthodontic need for the Peruvian population. The use of indexes validated elsewhere have to be considered carefully since the definition of a population's need is not universal. This is especially true for aesthetic perception of need.

## Methods

The study sample compromised of 281 first-year university students who were randomly selected from a population of 780 students in a private university from Lima (Peru). The registration list of the students admitted to the 2003 academic year was used as a sampling frame for the sample selection. Students with a past history or active orthodontic treatment were excluded from the study. The sample size was calculated for estimating an orthodontic treatment need of 24% in the population (using the DHC of the IOTN), at the 5% level (α = 0.05) and with a maximum tolerable error of 5%.

A structured face-to-face interview was carried out before the respective clinical examination of each student. First, students were asked to give their personal data as well as state their university tuition fee scale as an indirect measure of SES. In this university, students pay different tuition fees according to a socio-economic evaluation corroborated by a university social worker. An ordinal scale of three categories was available (low, medium and high SES). No additional information about desire and self-perceived need was requested from the students.

Thereafter, students rated their own perceived dental attractiveness on the AC of IOTN [6,12], and whereas the normative orthodontic treatment need was assessed according to the DHC of the IOTN [6]. During clinical examination, hypodontia was determined as the absence of at least one tooth in any quadrant with restorative implications and impacted tooth was determined as the impeded eruption of any tooth (disregarding third molars) due to occlusal or pathological causes [6].

Clinical examinations were carried out at the University Dental Clinic by one examiner with experience in epidemiological evaluation of orthodontic treatment need [28-31]. To minimize random and systematic errors, intra-examiner reliability was assessed through duplicated assessments for ten students on different days (0.89, weighted Kappa).

Statistical analyses were conducted using the statistical package Intercooled Stata 8.0 for Windows (Stata Corporation, Texas, USA). Both components of IOTN were determined in percentages separately. Chi-square test was used to determine if there were significant differences in the distribution of DHC and AC grades according to sex, SES and age of students. Median age was used to separate the students into two age groups, where one group was < 18 years and the second group was ≥ 18 years of age. The level of significance was set at 0.05.

## Results

The sample study (n = 281) consisted of 157 males (55.9%) and 124 females (44.1%). The mean age of the evaluated students was 18.1 +/- 1.6 years, with 79.3% ranging from 17 to 19 years old. The percentage distribution of students according to SES was 51.2%, 40.6% and 8.2% corresponding to low, medium and high SES respectively.

The objectively determined DHC distributions showed that 29.9% of the students were in great need of treatment (grades 4 and 5), 34.9% in moderate need of treatment (grade 3), and 35.2% with slight or no need for treatment (grades 2 and 1). Dental crowding, increased overjet and hypodontia were, in that order, the most common occlusal traits contributing to DHC grades (Table 1), with 57.2%, 12.4% and 6.4% of the evaluated students, respectively, having these traits.

**Table 1:** Distribution of the DHC grades in the sample of evaluated students

**DHC grades**	**n**	**Individual percentage**	**Overall percentage**
*Grade 5 (Definite need)*			9.3
5a	9	3.2	
5h	5	1.8	
5i	8	2.8	
5p	2	0.7	
5s	2	0.7	
*Grade 4 (Need)*			20.6
4a	8	2.8	
4d	33	11.7	
4f	2	0.7	
4h	13	4.6	
4l	2	0.7	
*Grade 3 (Borderline need)*			34.9
3a	18	6.4	
3d	77	27.4	
3e	1	0.4	
3f	2	0.7	
*Grade 2 (Slight need)*			27.4
2b	1	0.4	
2c	13	4.6	
2d	51	18.1	
2f	2	0.7	
2g	10	3.6	
*Grade 1 (No need)*			7.8
1	22	7.8	
*Total*	281	100.0	100.0

Table 2 shows the comparison of DHC grades according to the evaluated covariables. When DHC grades were compared by sex, age and SES of the students no statistically significant differences were found (*P *= 0.403, 0.543 and 0.247 respectively).

**Table 2:** Comparison of the DHC grades by sex, socio-economic status and age of the students

**Covariables**	**Definite need**		**Borderline need**		**No need**		***P *value**
		
	**n**	**%**	**n**	**%**	**n**	**%**	
*Sex*							0.403
Female	34	27.4	41	33.1	49	39.5	
Male	50	31.8	57	36.3	50	31.8	
*Age*							0.543
< 18 years	36	29.0	40	32.3	48	38.7	
≥ 18 years	48	30.6	58	36.9	51	32.5	
*Socio-economic status*							0.247
Low	50	34.7	42	29.2	52	36.1	
Medium	29	25.4	47	41.2	38	33.3	
High	5	21.7	9	39.1	9	39.1	

Chi-square test was used

Degree of freedom = 2 for sex and age, 4 for SES

The frequency distribution of AC of IOTN is exhibited in Figure 1. Only 1.8% of the students perceived themselves in definite need of treatment (photos 8–10), 11.0% in borderline need for treatment (photos 5–7) and 87.2% in no need for orthodontic treatment (photos 1–4). Photo number 2 was the most selected (30.6%).

**Figure 1:**
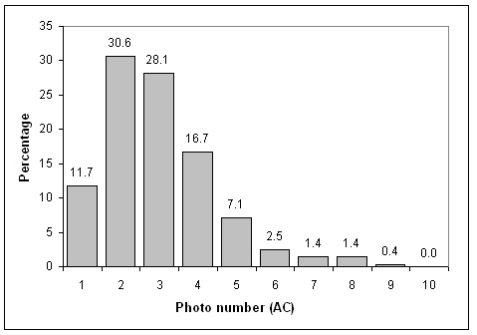
Distribution of AC grades in the sample of evaluated students.

Due to the limited number of cases which were self-perceived as definitely needing orthodontic treatment, categories of borderline need and definite need were collapsed to develop a more appropriate statistical analysis. (Table 3) No statistically significant differences for AC grades according to gender, age and SES of the students were found (*P *= 0.750, 0.750 and 0.054 respectively).

**Table 3:** Comparison of the AC grades by sex, socio-economic status and age of the students

**Covariables**	**Borderline or definite need**		**No need**		***p *value**
		
	**n**	**%**	**n**	**%**	
*Sex*					0.750
Female	15	12.1	109	87.9	
Male	21	13.4	136	86.6	
*Age*					0.750
< 18 years	15	12.1	109	87.9	
≥ 18 years	21	13.4	136	86.6	
*Socio-economic status (SES)*					0.054
Low	24	16.7	120	83.3	
Medium	12	10.5	102	89.5	
High	0	0.0	23	100.0	

Chi-square test was used

Degree of freedom = 1 for sex and age, 2 for SES

## Discussion

Several reasons have been previously reported to support the assessment of orthodontic treatment need in young adults [14-16]. In the present study, these young adults were selected for two additional reasons: first, their higher capability for expressing opinions on dental appearance in comparison to younger age groups [32,33], and second, for their accessibility since young adults consistently attend university, where they could be evaluated simultaneously.

The study sample included students from a private university located in Lima, Peru. This university was selected by convenience, and the students recruited represented a highly selective group of young adults. As such, the results from this study would not be truly representative of the young adult population of Lima. Policy makers and public health dentists should interpret these results with caution in light of this limitation. Further studies would be needed to verify or complement the outcomes of this study.

According to the DHC assessment, almost a third of the evaluated sample was placed in grades 4 and 5, indicating great and very great orthodontic treatment need, respectively. Previous studies in young adults indicated that normative orthodontic treatment need ranged from 1.4% to 71.6% [15,17-28,33]; however, different indexes were used in the studies (Table 4). Some of these studies have even included young adults with a history of previous orthodontic treatment [18,20,21,25,27].

**Table 4:** Previous studies reporting frequency of definite need for orthodontic treatment in young adults

**Study**	**Place**	**n**	**Age**	**Index**	**Definite need**	**Patients with HPOT**
*Index for Orthodontic Treatment Need (IOTN)*						
Present study	Peru	281	16 – 25	AC	1.8	---
				DHC	29.9	---
Hassan (2006) [33]	Saudi Arabia	743	17–24	AC	16.1	---
				DHC	71.6	---
Soh & Sandham (2004) [15]	Asia	339 males	17 – 22	AC	29.2	---
				DHC	50.1	---
Kerosuo et al (2004) [25]	Kuwait	139	14 – 18	AC	1.4	Included
				DHC	28.1	Included
Klages et al (2004) [27]	Germany	148	18 – 30	AC	0.0	Included
Kerosuo et al (2000) [23]	Finland	281	18 – 19	AC	0.0	---
				DHC	12.8	---
Tuominen et al (1995) [21]	Finland	89	16 – 19	DHC	11.2	Included
*Other Indexes for Orthodontic Treatment Need*						
Bernabé & Flores-Mir (2006) [28]	Peru	267	16 – 25	DAI*	32.6	
Baca-Garcia et al (2004) [26]	Spain	744	14 – 20	DAI	21.1	---
Onyeasco et al (2003) [24]	Nigeria	104	16 – 25	DAI	44.2	---
Stenvik et al (1996) [22]	Norway	50	18	NOTI**	0.0	---
Searcy & hisick (1994) [20]	Sweden	576 males	18 – 24	TPI*	16.3	Included
Espeland et al (1993) [18]	Norway	100	18	NOTI	8.0	Included
Espeland et al (1993) [19]	Norway	144	17 – 18	NOTI	1.4	---

HPOT = History of previous orthodontic treatment

* Only 'severe malocclusion' and 'handicapping malocclusion' are presented

** Only 'great need' and 'very great need' are presented

When only those studies using the DHC were compared, the frequency of treatment need in the present population was higher than those reported for Finns [21,23] and Kuwaiti citizens [25], but smaller than that reported for male Asians [15] and Saudi citizens [33]. (Table 4) Different selected sample sizes and age ranges within the evaluated young adults might have contributed to the reported differences in normative orthodontic treatment need.

In Peru, a previous study [28] carried out with the same population of first-year university students used the Dental Aesthetic Index (DAI). Despite the broadly discussed differences between both indexes in relation to the occlusal traits included in the assessment [3,4,11,34,35], the frequency of students needing orthodontic treatment was very similar in both studies (32.6% versus the 29.9% reported here).

Dental crowding, increased overjet and hypodontia were the most frequently scored occlusal traits. Although the overall frequency of students presenting with dental crowding was 57.2%, a displacement of teeth greater than 4 mm was only found in 11.7% of the sample, which indicates a need for orthodontic treatment (grade 4). Therefore, the remainder of the students only had slight dental crowding (≤ 4 mm), which was classified as not having a significant need for orthodontic treatment. Similarly, only 6.0% of the students- and not 12.4%-presented an increased overjet greater than 6 mm, which indicated a definite treatment need (grades 4 and 5). The remaining 6.4% presented with a mildly increased overjet (>3.5 mm but ≤ 6 mm). Compared with these adjusted results, the frequency of students with hypodontia requiring pre-restorative orthodontics was high (6.4%). This would mean that increased overjet is the third most common occlusal trait for definite treatment need.

The present findings agree with those previously reported by Hassan [33], who found that dental crowding was the predominant occlusal trait in Kuwaiti citizens. In addition, Bernabé and Flores-Mir [28], using the DAI index, found that in the same population of Peruvian students malocclusion was characterized by a relative high frequency of missing teeth, significant dental crowding and inadequate posterior occlusal relationships. On the contrary, Kerosuo et al [23] and Soh and Sandham [15] have reported that dental crossbite and crowding, in that order, were the most common occlusal traits scoring for definite treatment need in Finnish and Asian young adults respectively.

When self-perceived orthodontic treatment need was evaluated by means of the AC of IOTN, only a few of the first-year students (1.8%) self-scored as presenting a definite need for orthodontic treatment (photos 8–10). This corroborates the presence of a skewed distribution toward the attractive end of the scale as has been reported in previous studies [23,25,27,33].

A marked difference between normative (29.9%) and self-perceived (1.8%) treatment need in this population was detected. A possible explanation for which normatively defined need for orthodontic care was not matched by the perceived need is that the IOTN is a normative measure of something that is subjectively defined (aesthetics). Such a difference is supported by the conceptual distinction between health and disease [36]; while clinical indicators measure disease, which is a purely a biological concept, subjective indicators concentrate on health, a concept inclined more towards sociology and psychology [37].

Disease does not always negatively affect subjective perceptions of well-being, and even when it does, its impact depends on expectations, preferences, material, social and psychological resources and, more importantly, socially and culturally derived values [36,37]. What is considered aesthetically pleasing in one culture will often not match that which is thought of as aesthetically pleasing in another. Thus the lack of perceived need in the population evaluated might be due to the fact that Peruvian students probably do not have the same notions of beauty as their British peers, where the index was developed.

The level of education may also be a factor influencing treatment need and demand. The present results were based on highly educated individuals, which might not be truly representative of the general young adult population. Further studies should assess the perception of malocclusion and the level of orthodontic awareness in children, adolescents and young adults in addition to treatment need, to provide more precise information for manpower planning for the delivery of orthodontic care.

Another possible explanation could be the low frequency of orthodontic treatment requested by the Peruvian population. In Peru, the orthodontist to population ratio is very low, approximately 1/450,000 in Lima, and completely delivered by the fee-for-service modality [28]. The availability of orthodontic services has been shown to affect self-satisfaction of dental appearance and the desire of treatment in young adults [15,38]. Furthermore, Espeland et al [18,19] have reported that untreated young adults living in areas with low orthodontic treatment frequency were generally less aware of their anterior occlusal traits in comparison to young adults in areas with a higher treatment frequency. Presumably, different norms for acceptable dental arrangement operate in both areas [19].

In the present study sex, age and socio-economic status of the Peruvian first-year university students did not influence normative or self-perceived orthodontic treatment need. According to our literature review, the role of these factors in treatment need varies. Although sex seems to be the most studied covariable, findings still are contradictory. In a previous study, males were assessed by professionals as having need for treatment significantly more frequently than females [23], but others do not support this difference [21,25]. Also, some studies have shown that females are more selective in their self-perception than males, generally valuing dental appearance higher than males [2,39].

Stenvik et al [22] found that dissatisfaction with dental appearance and desire for orthodontic treatment decreased with increasing age, but more studies are required to assess age-related changes, which should be conducted longitudinally. The absence of differences in the distribution of normative and self-perceived need for orthodontic treatment according to socio-economic status is in agreement with some authors [25,40]; however, several other studies have reported differences between different socio-economic statuses [41, 42]. It is possible that a standardization of the criteria used to define social class will be needed before a summary of the different findings could be made [11].

According to the present findings, a third of the Peruvian first-year university young adults should receive orthodontic treatment to avoid the associated health risks generated by malocclusions [40], unfortunately not many of them have access to orthodontic treatment. One possible explanation may be that orthodontic concern is still given low priority in the oral health care system in Peru. Although there is a public health system, the lack of resources makes the funds available for dentistry scarce. Thus, orthodontic services are not readily available and accessible to the general population.

In that context, the current findings could be useful to plan orthodontic services for this specific university population, where an oral health insurance program exists including basic restorative treatment. Nevertheless, cost-benefit and cost-effectiveness analyses should be carried out first to assess the suitability of such a service.

In summary, further studies are required to improve our understanding of normative and self-perceived need for orthodontic treatment, especially in developing countries where the low frequency of orthodontic care added to the almost 100% private delivery of orthodontics have a significant influence. Hence, different factors than those reported in North American and European countries could be influencing the demand and delivery of orthodontic care. It may even be necessary to use more than one index in an epidemiological study to gather all the required information.

## Conclusion

• Approximately one-third of the evaluated Peruvian first-year university students presented a normative definite need.

• Only 1.8% self-perceived a need for orthodontic treatment need.

• Dental crowding greater than 4 mm, hypodontia, and increased overjet greater than 6 mm were the main reasons for determining orthodontic treatment need.

• Gender, age and socio-economic status of the students did not influence the frequency distribution of normative and self-perceived orthodontic treatment need.

## Competing interests

The author(s) declare that they have no competing interests.

## Authors' contributions

EB performed the statistical analysis and participated in the conception and draft of the manuscript.

CF conceived the study, participated in the study design, helped with the data collection, and coordinated and helped to draft the manuscript.

All authors read and approved the final manuscript.
